# Expression of phosphodiesterase 6 (PDE6) in human breast cancer cells

**DOI:** 10.1186/2193-1801-2-680

**Published:** 2013-12-18

**Authors:** Hongli Dong, Kevin P Claffey, Stefan Brocke, Paul M Epstein

**Affiliations:** Departments of Cell Biology, University of Connecticut Health Center, 263 Farmington Ave, Farmington, CT 06030-3505 USA; Immunology, University of Connecticut Health Center, Farmington, CT 06030 USA

**Keywords:** Breast cancer, Cyclic nucleotide phosphodiesterase, PDE6, Light, Circadian clock genes, cGMP signaling

## Abstract

Considerable epidemiological evidence demonstrates a positive association between artificial light at night (LAN) levels and incidence rates of breast cancer, suggesting that exposure to LAN is a risk factor for breast cancer. There is a 30-50% higher risk of breast cancer in the highest LAN exposed countries compared to the lowest LAN countries, and studies showing higher incidence of breast cancer among shift workers exposed to more LAN have led the International Agency for Research on Cancer to classify shift work as a probable human carcinogen. Nevertheless, the means by which light can affect breast cancer is still unknown. In this study we examined established human breast cancer cell lines and patients’ primary breast cancer tissues for expression of genetic components of phosphodiesterase 6 (PDE6), a cGMP-specific PDE involved in transduction of the light signal, and previously thought to be selectively expressed in photoreceptors. By microarray analysis we find highly significant expression of mRNA for the *PDE6B*, *PDE6C*, and *PDE6D* genes in both the cell lines and patients’ tissues, minimal expression of *PDE6A* and *PDE6G* and no expression of *PDE6H*. Using antibody specific for PDE6β, we find expression of PDE6B protein in a wide range of patients’ tissues by immunohistochemistry, and in MCF-7 breast cancer cells by immunofluorescence and Western blot analysis. Considerable expression of key circadian genes, *PERIOD 2, CLOCK, TIMELESS, CRYPTOCHROME 1,* and *CRYPTOCHROME 2* was also seen in all breast cancer cell lines and all patients’ breast cancer tissues. These studies indicate that genes for PDE6 and control of circadian rhythm are expressed in human breast cancer cells and tissues and may play a role in transducing the effects of light on breast cancer.

## Introduction

Cyclic Nucleotide Phosphodiesterases (PDEs) are encoded by 21 different genes grouped into 11 gene families based on sequence similarity, mode of regulation, and specificity for cAMP or cGMP as substrate (Francis, et al. 2011; Lerner and Epstein 2006). There is considerable interest in PDEs owing to studies showing them to be excellent candidate targets for treating a wide range of illnesses including hematological malignancies, osteoporosis and inflammatory and autoimmune diseases (Epstein 2012; Francis, et al. et al. 2011; Lerner and Epstein 2006; Vang, et al. et al. 2010). Catalytic subunits of PDE6, a cGMP-specific PDE, were thought to be photoreceptor-specific, localized exclusively to the outer segment of rods and cones in the eye, where they are crucial for the transduction of light (Stryer 1986; Zhang and Cote 2005). The rod outer segment (ROS) is a dual membrane system composed of about 2000 disks surrounded by a physically separate plasma membrane. The disks contain rhodopsin, PDE6 and transducin. The plasma membrane contains a cGMP-gated sodium channel. In the dark, the ROS contains an unusally high concentration of cGMP (≈ 70 μM), which activates the sodium channel and depolarizes the membrane. When light impinges on rhodopsin on the disks, it photoisomerizes its chromophore, which in turn activates the GTP-binding protein, transducin, which activates the catalytic activity of PDE6 by dissociating its inhibitory γ subunits. PDE6 then very rapidly reduces the cGMP concentration leading to inactivation of the sodium channels and hyperpolarization of the plasma membrane which is conveyed to the synapse at the other end of the rod and communicated to other cells of the retina (Stryer 1986; Zhang and Cote 2005). In the human rod, PDE6 is composed of two large (≈99 kDa) catalytic subunits, α and β, encoded by the *PDE6A* and *PDE6B* genes respectively, two small (≈ 11 kDa) inhibitory γ subunits encoded by *PDE6G*, and a small (≈ 17 kDa) δ regulatory subunit encoded by *PDE6D*. The human cone PDE6 contains two α’ catalytic subunits encoded by *PDE6C*, two inhibitory γ subunits encoded by *PDE6H*, and a regulatory δ subunit encoded by *PDE6D* (Ionita and Pittler 2007). The *PDE6D*-encoded δ subunit (PDE6δ) is not specific to PDE6 in that it binds to other enzymes and is expressed in other tissues (Zhang, et al. 2004). Its function as part of the PDE6 enzyme is unclear, although it is generally found to be bound to the soluble fraction of PDE6 in retina extracts and may play a role in transport of PDE6 to the disks or its translocation to the plasma membrane (Cook, et al. 2001; Zhang, et al. et al. 2007). PDE6δ has also been shown to function as a cytosolic farnesyl-binding chaperone protein for ras, which facilitates ras trafficking and signaling (Chandra, et al. 2012). The γ inhibitory subunit of PDE6 is also expressed in other tissues such as lung and kidney cells (Guo and Ruoho 2008; Tate, et al. et al. 2002) and has been suggested to play a role in the activation of MAP kinase by epidermal growth factor signaling (Wan, et al. 2003). PDE6 catalytic activity has also been reported in chick pineal gland where it is believed to mediate the light induced inhibition of melatonin synthesis (Holthues and Vollrath 2004; Morin, et al. et al. 2001). In contrast to humans, in chick, both in rods and pineal glands, the catalytic activity of PDE6 appears to be comprised of homodimers of two *PDE6B*-encoded β subunits, with no evidence for the expression of *PDE6A*-encoded α subunits present in these chick tissues (Huang, et al. 2004; Morin, et al. et al. 2001). Inasmuch as cGMP has been shown to regulate proliferation and apoptosis of breast cancer cells (Saravani, et al. 2012; Tinsley, et al. et al. 2011), we examined breast cancer cells for the expression of PDE6 genes. In this paper we show significant expression of PDE6B, PDE6C, and PDE6D, in human breast cancer cell lines and patients’ breast cancer tissues.

## Materials and methods

### Materials

Breast cancer/uninvolved paired tissue array (catalog no. BRC481) was obtained from Pantomics (San Francisco, CA, USA). Tissues were classified as tumor or uninvolved based upon H&E staining and immunohistochemistry using anti-cytokeratin antibody and staged according to the standard TNM classification by a certified physician. Human breast adenocarcinoma estrogen receptor-positive MCF-7 and T-47D, and estrogen receptor-negative MDA-MB-231 and MDA-MB-435 cell lines were obtained from American Type Culture Collection (Manassas, VA, USA). Breast cancer tumor tissues for microarray analysis were obtained from the Neag Cancer Center of the University of Connecticut Health Center (UCHC) in a deidentified manner. All procedures involving human tissue were approved by the UCHC Institutional Review Board. PDE6β specific antibody (catalog no. NB120-5663) was from Novus Biologicals (Littleton, CO). Alexa Fluor 350 conjugated goat anti-rabbit IgG was from Invitrogen (Carlsbad, CA). Horseradish peroxidase conjugated goat anti-Rabbit IgG was from GE Healthcare (Piscataway, NJ).

### Cell culture

Cell lines were maintained in DMEM medium supplemented with 10% fetal bovine serum, 2 mM L-glutamine, 100 U/ml penicillin and 100 μg/ ml streptomycin, at 37°C in a humidified atmosphere of 95% air and 5% CO_2_.

### Microarray

Microarray analyses were performed with Illumina Ref8 bead chips according to the manufacturer’s directions. Data analysis was performed with Illumina Gene Studio software. Gene array data for cell lines or tumor samples were treated as separate groups and normalized using rank-invariant normalization of raw data. Statistical significance of each value was determined based upon signal variance for multiple probes for each gene to assume appropriate mRNA expression.

### Western immunoblot analysis

Western immunoblotting analysis was performed as described previously (Vang, et al. 2013). MB-231 and MCF-7 breast cancer cells were centrifuged at 300 × g for 5 min, washed twice with ice-cold phosphate buffered saline (PBS), and lysed in RIPA buffer (50 mM Tri-HCl, pH 7.4, 150 mM NaCl, 1 mM EDTA, 1% NP-40, 0.25% Na-deoxycholate with 1:100 dilution Sigma protease inhibitor cocktail). Protein concentration was determined using a Micro BCA Protein Assay Kit (Pierce, Rockford, IL). Equal amounts of protein were loaded and run on 10% SDS-PAGE gels. Proteins were then transferred onto Immobilon-p Transfer Membrane (Millipore). Membranes were blocked with 5% BSA in Tris-buffered saline (TBS) for 1 h at room temperature and probed with 1:200 dilution of PDE6β specific primary antibody (Novus Biologicals) overnight at 4°C, washed three times with TBS-T (TBS with 0.1% Tween 20) buffer, and incubated with horseradish peroxidase-conjugated secondary antibody (GE Healthcare) at 1:5000 dilution for 1 h at room temperature and then washed three more times. Proteins were visualized with SuperSignal West Femto Maximum Sensitivity Substrate (Pierce, Rockford, IL) using a Syngene G:Box with Genesnap BioImaging software. Staining with anti-GAPDH (glyceraldehyde-3-phosphate dehydrogenase) antibody (Abcam) was used for loading control and normalization.

### Immunofluorescence

MCF-7 and MDA-MB-231 cells were seeded on a slide cell chamber at 5×10^3^ cells/well. After growth for 24 h the cells were washed 3 times with PBS, fixed in 4% paraformaldehyde in PBS for 10 min and washed with PBS 3 times. The fixed cells were blocked with 5% BSA in PBS containing 0.2% Tween-20 at room temperature for 2 h, incubated with primary anti-PDE6β antibody (1:50) overnight at 4°C, washed 3 times and incubated with Alexa Fluor conjugated secondary antibody (1:200) for 1 h, washed 3 times in PBS, counterstained with 4',6-diamidino-2-phenylindole (DAPI) (1:5000 in PBS) for 2–5 min, washed 3 times and examined by fluorescent microscopy.

### Immunohistochemistry

Immunohistochemistry was performed on 4 μm thick paraffin sections from 16 cases of invasive breast cancer with corresponding uninvolved tissue obtained from Pantomics using primary PDE6β antibody and detection with Vector ImmPRESS anti-rabbit Ig (peroxidase) polymer detection kit using DAB substrate according to the manufactuer’s protocol (Vector Laboratories, Burlingame, CA, USA). Controls without addition of primary antibody were performed to assure signal specificity. Nuclei were counterstained using methyl green.

## Results

### Expression of PDE6 mRNA in breast cancer cell lines

**Figure 1:**
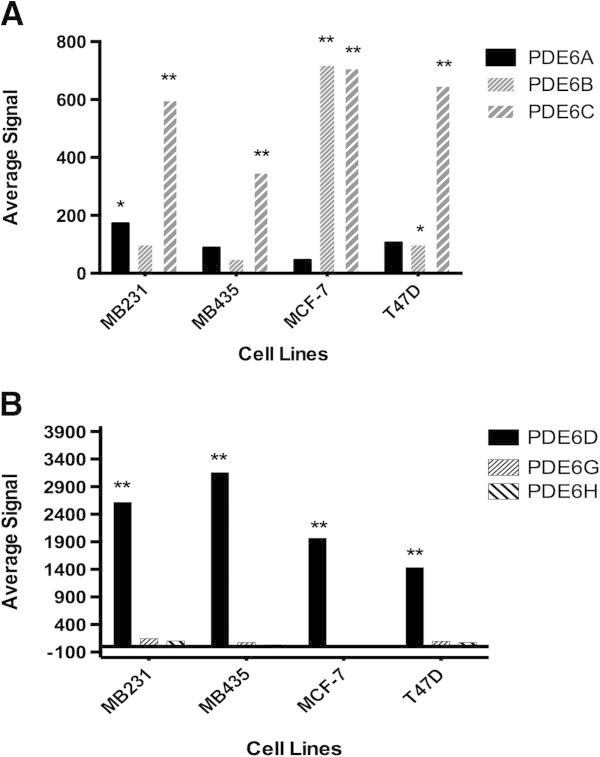
**Expression of PDE6 mRNA in breast cancer cell lines. (A)** Microarray analysis of PDE6 catalytic (*PDE6A*, *6B* and *6C*) subunit mRNA expression. **(B)** Microarray analysis of PDE6 regulatory (*PDE6D*) and inhibitory (*PDE6G* and *6H*) subunit mRNA expression. **P < 0.01 and *P < 0.05.

Expression of the mRNA for PDE6 genes was examined in the human breast adenocarcinoma estrogen receptor-negative cell lines, MB-231 and MB-435, and in the estrogen receptor-positive cell lines, T47D and MCF-7, by microarray analysis. Figure 1A shows the expression of mRNA for PDE6 catalytic subunit genes, *PDE6A*, *6B*, and *6C. PDE6B* was highly and significantly expressed in MCF-7 cells and to a much smaller extent in T47D cells, but not in MB-231 and MB-453 cells. *PDE6C* was highly and significantly expressed in all four of these breast cancer cell lines. *PDE6A* was minimally expressed at a significant level only in MB-231 cells, and not in any of the other breast cancer cells. Figure 1B shows the expression of mRNA for PDE6 regulatory subunit genes, *PDE6D*, *6G*, and *6H*. No significant expression of genes for the rod inhibitory subunit, *PDE6G*, or the cone inhibitory subunit, *PDE6H*, was seen in any of the cell lines examined. The regulatory subunit gene, *PDE6D*, was, however, highly expressed in all four breast cancer cell lines.

### Expression of PDE6 protein in breast cancer cell lines

**Figure 2:**
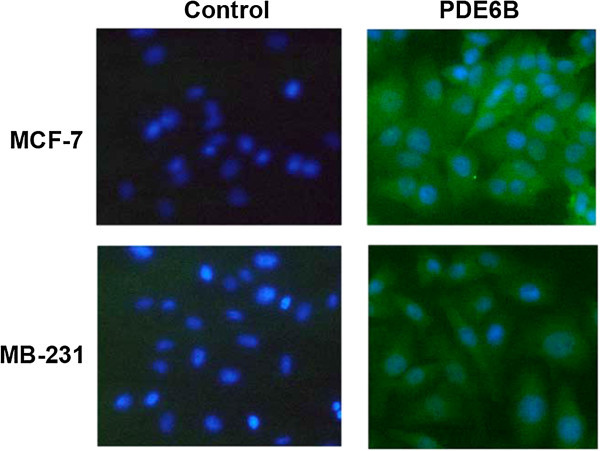
**Expression of PDE6B protein in breast cancer cell lines by immunofluorescence.** MCF-7 and MDA-MB-231 cell lines were stained with PDE6β primary and Alexa Fluor conjugated secondary antibodies and counterstained with DAPI as described in Methods. Controls were treated identically except that the primary antibody was omitted.

**Figure 3:**
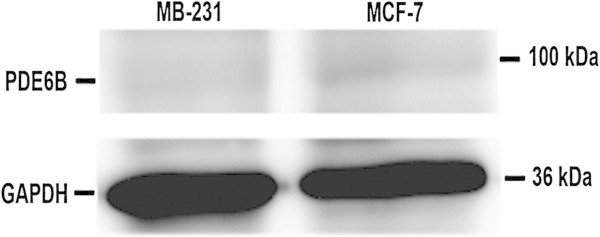
**Expression of PDE6B protein in breast cancer cell lines by Western immunoblot analysis.** Protein extracted from MCF-7 and MDA-MB-231 cell lines were run on SDS-PAGE gels, transferred to Immobilon-P membrane, probed with PDE6β specific antibody and detected with horseradish peroxidase conjugated goat anti-Rabbit IgG as described in Methods.

Using antibody specific for PDE6β, the expression of PDE6B protein was examined in MCF-7 and MB-231 cells both by immunofluorescence and Western immunoblot analysis. Consistent with *PDE6B* mRNA expression, as shown in Figure 2, by immunofluorescence, high expression of PDE6B protein was also seen in MCF-7 cells and the protein appeared to be expressed throughout the cell. Little expression of PDE6B protein was seen in MB-231 cells by immunofluorescence, again consistent with the mRNA expression. Also consistent with the *PDE6B* mRNA expression, as shown in Figure 3, PDE6β specific antibody recognized a band at ≈ 99 kDa in MCF-7 cells by Western immunoblot analysis, but little expression of this band was seen in MB-231 cells.

### Microarray analysis of PDE6 mRNA in patients’ breast cancer tissue

**Figure 4:**
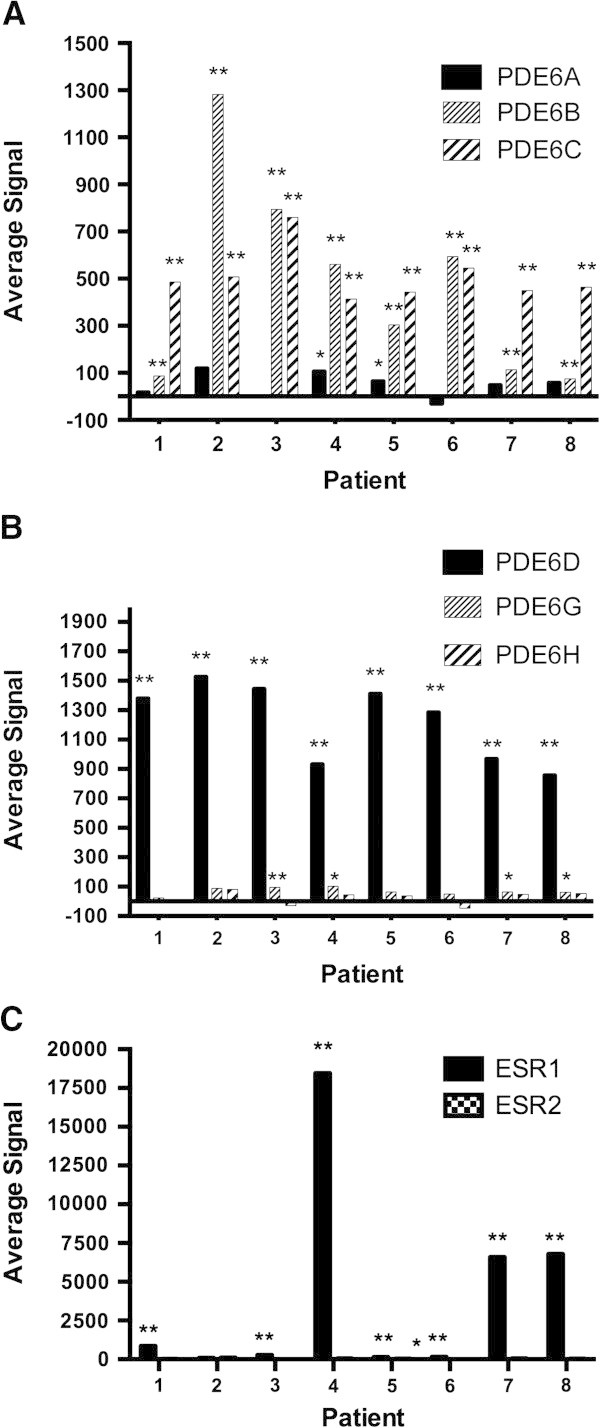
**Expression of PDE6 and estrogen receptor mRNA in patients’ breast cancer tissue.** Breast cancer tumor tissues from eight patients were analyzed by Illumina microarray analysis. **(A)** Microarray analysis of PDE6 catalytic (*PDE6A*, *6B* and *6C*) subunit mRNA expression. **(B)** Microarray analysis of PDE6 regulatory (*PDE6D*) and inhibitory (*PDE6G* and *6H*) subunit mRNA expression. **(C)** Microarray analysis of estrogen receptor alpha mRNA expression. **P < 0.01 and *P < 0.05.

PDE6 gene expression was analyzed by microarray analysis in breast tumor tissue from eight breast cancer patients. Similar to results with the breast cancer cell lines (Figure 1), in patients’ tumor tissues, as shown in Figure 4A, only very minimal expression of *PDE6A* was seen, which only reached significance in two of the eight patients, patient numbers 4 and 5. In contrast, *PDE6B* and *PDE6C* were significantly, and in many cases highly, expressed in all eight patients’ tumor tissues. Examination of the expression of the PDE6 regulatory genes, shown in Figure 4B, also closely paralleled that seen in the breast cancer cell lines in that only very minimal expression for *PDE6G* was seen in any tissues, and no significant expression was seen for *PDE6H* at all, but considerable and significant expression was seen for *PDE6D* in all eight patients’ breast tumor tissues. Estrogen receptor expression was also examined in these tissues to determine their estrogen receptor status. As shown in Figure 4C, significant expression of estrogen receptor α (*ESR1*) was seen in all patients’ samples except for patient number 2, although the degree of expression varied considerably from patient to patient. Based on these data, there does not appear to be any obvious correlation between expression of PDE6 genes and estrogen receptor status.

### Expression of PDE6 protein in breast cancer tumors

**Figure 5:**
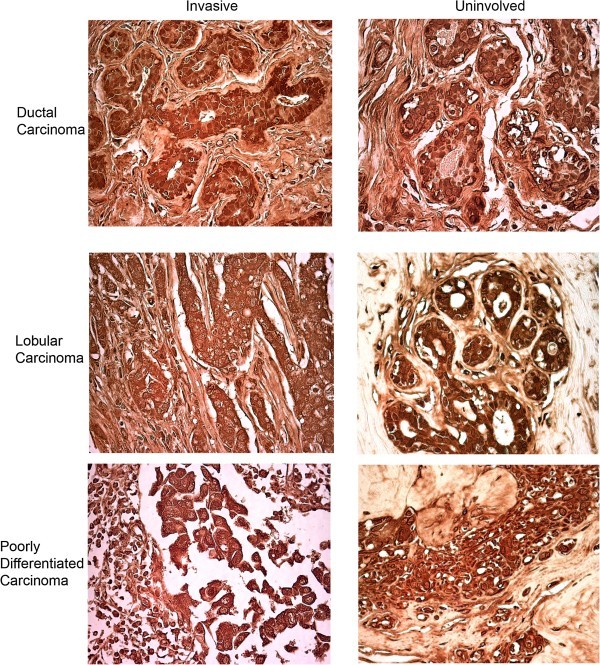
**Expression of PDE6B protein in patients’ breast cancer tissue.** Immunohistochemistry with PDE6β antibody was performed on paraffin sections from sixteen cases of invasive breast cancer, which included eleven cases of ductal, three cases of lobular, and two cases of poorly differentiated carcinoma, with corresponding uninvolved tissue. All sixteen tissue samples were positive for PDE6B expression and a representative sample of each of the three types of carcinoma with its corresponding uninvolved tissue is shown.

Using antibody specific for PDE6β, the expression of PDE6B protein was examined in sixteen cases of invasive breast cancer and surrounding tissue by immunohistochemistry. Considerable expression of PDE6B protein was seen in all sixteen breast cancer tissue samples, regardless of what type of breast carcinoma they represented. The sixteen samples contained eleven cases of ductal carcinoma, three cases of lobular carcinoma, and two cases of poorly differentiated carcinoma. PDE6B expression in a representative example of each of these types of cancer is shown in Figure 5. Considerable PDE6B expression is seen in all of these, and expression is seen in the uninvolved tissue surrounding these tumors as well. In controls where primary PDE6β antibody was omitted, no labeling was seen at all (not shown). Although the tissue samples surrounding the tumors are classified as uninvolved, these do not necessarily represent normal breast tissue, since they are in proximity with the tumor and thus may already be in a precancerous state, exhibiting changes such as non-cancerous atypia or hyperplasia.

### Expression of phototransduction genes, wnt signaling genes, and circadian clock genes in breast cancer cell lines and patients’ tumors

Bazhin et al. showed that in addition to PDE6, other proteins in the phototransduction pathway, including rhodopsin, transducin, cyclic nucleotide gated (CNG) channels, guanylyl cyclase, rhodopsin kinase, recoverin and arrestin, are also expressed to varying degrees, although all to a lesser extent than PDE6, in melanoma cell lines and tissues, and they refer to these expressed genes as cancer-retina antigens (Bazhin, et al. 2007). It was also shown that PDE6 is expressed in murine F9 teratocarcinoma cells (Ahumada, et al. 2002) and that PDE6 in these cells as well as in melanoma cells (Bazhin, et al. 2010) is activated by the noncanonical Wnt signaling pathway through Wnt5a binding to Frizzled-2 receptors, leading to activation of transducin and subsequent activation of PDE6. The result of this is a reduced concentration of cGMP and increased concentration of Ca^2+^. Additional studies have shown that expression of key circadian clock genes are under the control of cGMP (Golombek, et al. 2004; Oster, et al. et al. 2003; Plano, et al. et al. 2012), and that disruption of the normal expression of these genes can result in the development of cancers, including breast cancer (Fu, et al. 2011; Fu and Lee 2003; Gery and Koeffler 2010; Hoffman et al. 2010a,b). For these reasons, we also examined the expression of other phototransduction pathway genes, Wnt signaling genes, and circadian genes in breast cancer cells and tissues. As shown in Table 1, examination of phototransduction genes in breast cancer cells and tissues reveals highly significant expression (p < 0.01) of various isoforms of transducin, in addition to PDE6, but only minimal and sporadic expression of any of the other genes in the phototransduction pathway. Significant expression of *WNT5A* was observed in all of the patients’ breast cancer tissues and in the MB-435 and MB-231 cells, but not in the MCF-7 and T47D cells. *FRIZZLED-2* (FZD2) was significantly expressed in all breast cancer cell lines and tissues examined. The key circadian genes, *PERIOD 2* (*PER2* variant 2), *CLOCK*, *TIMELESS*, *CRYPTOCHROME 1* (*CRY1*) and *CRYPTOCHROME 2* (*CRY2*) were all also significantly expressed in all breast cancer cell lines and all patients’ breast cancer tissues examined.

**Table 1:** **Expression of phototransduction pathways genes, Wnt genes, and Circadian rhythm genes**

		Breast cancer cell lines (Average signal)				Patients’ breast cancer tissues (1-8) (Average signal)							
		MCF-7	T47D	MB-435	MB-231	1	2	3	4	5	6	7	8
Phototransduction genes													
RHO	Rhodopsin	-45	27	31	87	-43	40	-63	14	-45	-90	13	-1
GRK1	Rhodopsin kinase	4.25	102	70	133.9								
GNAT1 variant 1	Rod transducin alpha subunit	0	77	69	133	8	85	-12	54	31	4*	36	46
GNAT1 variant 2	Rod transducin alpha subunit	73	131*	90	124	54*	108	47**	63	60**	-7	75*	59
GNAT2	Cone transducin alpha subunit	27	93	83	248**	23	172**	-13	26	84**	-29	47	40
GNAZ	Cone transducin alpha subunit	199**	84	204**	118	139*	132**	131**	80**	255**	8*	250**	301
GNB1	Transducin beta subunit	22340**	14936**	14239**	17968**	20122**	20994**	17661**	15477**	21669**	19486**	20140**	17137**
GNB1L	Transducin beta subunit	925**	265**	1160**	1352**	607**	900**	1030**	261**	295**	749**	270**	343**
GNB2	Transducin beta subunit	2968**	675**	1208**	741**	1456**	691**	1171**	773**	3845**	1147**	1066**	1038**
GNG2L	Transducin beta subunit	69029**	73280**	43084**	56176**	62765**	50370**	70951**	65617**	74326**	62703**	59073**	25580**
GNB3	Transducin beta subunit	87*	95	136**	118	111*	117*	50**	179**	126**	23**	76**	96
GNB4	Transducin beta subunit	29	2157**	831**	1250**	229**	204**	687**	186**	288**	702**	565**	591
GNB5 variant 1	Transducin beta subunit	37	58	374**	217**	62**	85	58**	61	64**	17**	69	58
GNB5 variant 2	Transducin beta subunit	-7	54	31	106	19	73	-29	26	13	-61	7	41
GNGT1	Transducin gamma subunit	60	102	63	113	17	89	-24	63	25	-33	40	31
GNGT2	Transducin gamma subunit	-60	64	31	68	7	115	183**	74	15	93**	41	38
SAG	Arrestin	1	85	67	107	33	99	-44	107**	-1	14**	56	51
RLV1	Recoverin	-25	49	59	77	-21	63	7	60	32	-26	19	23
CNGA1	CNG channel alpha subunit	9	64	45	253	16	53	137**	10	139**	-50	30	15
CNGA2	CNG channel alpha subunit	3	76	65	97	4	71	-42	41	31	-41	49	33
CNGA3	CNG channel alpha subunit	-42	5	41	80	-29	66	-64	70	-30	-83	131**	145**
CNGB1	CNG channel beta subunit	13	23	27	81	6	74	-34	19	-7	-35	15	17
CNGB3	CNG channel beta subunit	14	43	44	95	10	93	-51	53	-18	-57	37	13
Wnt genes													
WNT5A	Wnt	-31	10	293**	113	915**	670**	84**	490**	775**	266**	195**	267**
FZD2	Frizzled-2	864**	1740**	1828**	4896**	1213**	220**	1045**	1576**	1088**	620**	1176**	1186**
Circadian rhythm genes													
PER1	Period 1	-25	69	53	80	32	100	-23	64	25	-1	62	102**
PER2 variant1	Period 2	7	56	59	93	-6	72	-31	32	21	-48	41	8
PER2 variant2	Period 2	5176**	2856**	1169**	846**	3847**	2745**	2693**	3234**	4415**	3693**	4735**	4683**
PER3	Period 3	4	64	49	89	6	73	-36	33	43	-12	77*	47
CLOCK	Clock	208**	330**	175**	216**	1001*	202*	364**	352**	575**	353**	468**	411*
TIMELESS	Timeless	5334**	8868**	3219**	4254**	2094**	1527**	1478**	2427**	1167**	2119**	1836**	1603**
CRY1	Cryptochrome 1	1302**	688*	682**	2693**	402**	1071**	474**	707**	326**	1205**	1052**	1166**
CRY2	Cryptochrome 2	2862**	1014**	494**	334**	1058**	908**	1622**	1611**	2053**	1604**	1171**	1078**

**P<0.01, *P<0.05, Significant values according to statistical variation for each gene probe analyzed on the Illumina array.

## Discussion

These studies demonstrate that PDE6, formerly thought to be photoreceptor-specific, is expressed in breast cancer cells and tissues both at the mRNA and protein level. In contrast, microarray analysis of PDE6 in completely normal tissue, using a murine system, shows PDE6 expression to be restricted to photoreceptor tissue, with no evidence at all of any PDE6 expression in normal breast epithelium (Laule, et al. 2006). Several other studies have also detected expression of PDE6 in non-photoreceptor tissues; specifically, in murine F9 embryonic teratocarcinoma cells, in melanoma cells, and in human lung tissue (Ahumada, et al. 2002; Bazhin, et al. et al. 2010; Nikolova, et al. et al. 2010). In F9 teratocarcinoma cells PDE6 is activated by Wnt5a-Frizzled-2 signaling acting through transducin, which is also expressed in these cells, and inhibition of PDE6 with isobutylmethylxanthine, dipyridamole or zaprinast inhibits the formation of primitive endoderm induced by retinoic acid in these cells, suggesting a role for PDE6 in early embryonic development (Ahumada, et al. 2002). PDE6 as well as all the other components of the visual phototransduction pathway were found to be expressed both in normal melanocytes and melanoma cells at the mRNA level, but only in melanoma cells at the protein level (Bazhin, et al. 2007). Some melanoma cell lines expressed Wnt5a and Frizzled-2, and in these cell lines Wnt5a stimulated PDE6 activity, suggesting that in melanoma, PDE6 may be activated either by light impinging on rhodopsin, which is expressed in these cells, or by Wnt5a acting through Frizzled-2 and transducin (Bazhin, et al. 2010). A functional role for PDE6 in melanoma cells is still not known. PDE6 catalytic subunit mRNA and protein were found to be expressed in both normal and fibrotic human lung tissue, and at comparable levels, but expression of the PDE6D regulatory and PDE6G/H inhibitory subunits were significantly reduced in fibrotic lung tissue. Overexpression or suppression of PDE6D expression led to changes in cGMP levels, rate of proliferation, and state of ERK phosphorylation, suggesting that alterations in PDE6D expression and its consequent effects on PDE6 activity may be a causative factor for idiopathic pulmonary fibrosis (Nikolova, et al. 2010).

Considerable epidemiologic evidence has been amassed over the past 25 years demonstrating an association between increased exposure to LAN and increased rates of breast cancer (Flynn-Evans, et al. 2009; Kloog, et al. et al. 2010; Stevens 2009a,b). Specific evidence for this includes increased incidence of breast cancer among women who work non-day shifts, lower incidence of breast cancer among totally blind women, an inverse relationship between breast cancer incidence and sleep duration, and a correlation between increased incidence of breast cancer and population LAN levels worldwide. This epidemiological evidence led the International Agency for Research on Cancer to classify shift work as a probable human carcinogen (Straif, et al. 2007). The molecular basis for effects of LAN on breast cancer are unknown, but inasmuch as the synthesis and secretion of melatonin from the pineal gland at night is very sensitive to suppression by LAN (Vanecek 1998), specifically only blue light in the wavelength range of 450–480 nm (Sanchez-Barcelo, et al. 2012), the general thinking has been that the reduced melatonin may be permissive for increased proliferation of breast epithelial cells and may thus play a role in the increased incidence of breast cancer (Sanchez-Barcelo, et al. 2012). Attention has also focused recently on the possibility that LAN exposure may affect circadian rythym through altering the expression of circadian genes and that this disruption of the circadian rhthym contributes to breast cancer development (Gery and Koeffler 2010; Savvidis and Koutsilieris 2012; Stevens 2009a, b). The body’s circadian rythym is normally regulated by the expression of circadian genes in a master pacemaker in the suprachiasmatic nucleus of the hypothalamus that signals to oscillators in peripheral tissues (Savvidis and Koutsilieris 2012). As shown in this paper (Table 1), as well as by others (Fu, et al. 2011; Hoffman et al. 2010a,b), circadian genes are expressed directly in breast cancer epithelial cells as well. Considerable evidence shows that expression of circadian genes are directly under the regulation of cGMP (Golombek, et al. 2004; Oster, et al. et al. 2003; Plano, et al. et al. 2012); thus, regulation of PDE6 in breast cancer cells by light or by Wnt5a signaling through Frizzled-2 and transducin, all of which we find expressed in breast cancer cells, could directly regulate the expression of circadian genes in these cells through alterations in cGMP levels. Although we find no evidence of rhodopsin in breast cancer cells to act as a light sensor, we do find high epression of CRY2 in these cells, and CRY2 can also act as a light sensor, absorbing light in its associated FAD molecule, intriguingly also specifically in the blue wavelength region of 450–480 nm (Hoang, et al. 2008), which may contribute to the activation of PDE6 in these cells, and the alteration in cGMP levels, leading to dysregulation of circadian genes, alterations in circadian rythyms, and the inducement or perpetuation of the growth of breast cancer cells. It is also noteworthy that increased cGMP levels have been shown to inhibit proliferation and induce apoptosis in breast cancer cell lines (Saravani, et al. 2012; Tinsley, et al. et al. 2011) and it is thus possible that breast cancer cells have evolved to express PDE6 as a means to protect themselves against cGMP-mediated apoptosis to ensure their continued growth. While there is still much to be done to decipher the true function(s) for the expression of the light-activated PDE6 in breast cancer cells, this study nevertheless documents the expression of PDE6 in these cells, thereby uncovering a potential novel means by which exposure to LAN may result in breast cancer growth and development.

## Conclusions

This paper demonstrates the novel finding of the expression of PDE6, the light sensitive PDE, previously thought to be photoreceptor specific, in breast cancer cell lines and patients’ breast cancer tissues, at both the RNA and protein levels. This finding is discussed in the context of epidemiological evidence for exposure to artificial light at night as a risk factor for breast cancer and in relation to our further finding of the expression of circadian rhythm genes in these breast cancer tisssues.

## Abbreviations

CNG: Cyclic nucleotide gated channels
CRY1: Cryptochrome 1
CRY2: Cryptochrome 2
DAPI: 4',6-diamidino-2-phenylindole
ESR: Estrogen receptor
FZD2: Frizzled-2
GAPDH: Glyceraldehyde-3-phosphate dehydrogenase
LAN: Artificial light at night
PBS: Phosphate buffered saline
PDE6: Phosphodiesterase 6
PER2: Period 2
ROS: Rod outer segment
TBS: Tris-buffered saline
UCHC: University of Connecticut Health Center.
